# A New Scheme of Applying CORPS and Crossovers to Reduce the Number of Phase Shifters in Antenna Arrays

**DOI:** 10.3390/s22218207

**Published:** 2022-10-26

**Authors:** Gilberto Calvillo, Marco A. Panduro, Brian Sanchez, Alberto Reyna

**Affiliations:** 1 CICESE Research Center, Electronics and Telecommunications Department, Carretera Ensenada-Tijuana No. 3918, Zona Playitas, Ensenada 22860, Baja California, Mexico; 2 Universidad Autónoma de Tamaulipas, UAMRR-R, Carretera Reynosa-San Fernando, Reynosa 88779, Tamaulipas, Mexico

**Keywords:** linear array, scanning range, phase shifter, CORPS

## Abstract

This paper presents a new scheme of applying CORPS (coherently radiating periodic structures) for reducing the number of phase shifters in linear antenna arrays. This scheme can be seen as a combination of the properties of two techniques: CORPS and butler. The proposed system applies an interleaving of several blocks of 2 × 3 CORPS networks. This interleaving of two stages of 2 × 3 CORPS networks is made in a convenient way to provide the required progressive phase for beam-scanning and the level of amplitude excitations necessary for achieving the radiation characteristics of low SLL. Interesting results are provided based on experimental measurements and full-wave simulations to analyze and evaluate the performance of the feeding network based on CORPS and the reduction capability of the number of phase shifters in the antenna system. The proposed design methodology achieves a reduction capability of 66% in the number of phase shifters used in linear antenna arrays. This reduction in the complexity of the antenna system is reached maintaining a peak SLL of −22 dB with scanning ranges of until ±25°. A good design option is provided to simplify the complexity of the feeding network in antenna array applications.

## 1. Introduction

The new sensor technologies require novel radiating systems with better performance characteristics. Antenna arrays play a very important role as radiating systems in the new generation of communication systems. New communications systems can be possible with the application of different structures of antenna arrays based on beam-forming networks [1]. These beam-forming networks are responsible of generating different beams in certain directions of a scanning range with the desired characteristics of the side lobe level (SLL). The complexity of these beam-forming networks to generate desired patterns limit the application of antenna arrays in communication systems. The applications of antenna arrays will increase with key benefits for the system if the complexity of the beam-forming networks is decreased. Simpler, low-cost beam-forming networks that provide the required performance are required. One way of reducing the complexity of the beam-forming networks is to reduce the number of phase shifters used in the antenna system [2].

There are several design methodologies in the literature for generating beam-forming networks. There is an interesting study line from the traditional (or classical) networks such as the butler matrix [3], Blass [4], and Nolen [5] to the most recent feeding networks for reducing the number of active devices including the application of subarrays [6,7,8,9,10,11,12,13], overlapping techniques [14,15,16,17,18], interleaving schemes [19] and CORPS (coherently radiating periodic structures) networks [20,21,22,23].

The use of subarrays is the most used technique to reduce the number of phase shifters. The antenna array can be partitioned in groups of uniform subarrays [11,13] or non-uniform subarrays [1]. A high number of phase shifters can be reduced by using subarrays. However, the radiation characteristics of array systems deteriorate substantially. So, the design problem complexity increases as the subarray size is increased. The overlapping [14] and interleaving [19] techniques could provide a reduction in the number of phase shifters and generate acceptable radiation characteristics. However, these techniques in general are complex and not easy to fabricate in the feeding network system.

It has been demonstrated recently that the CORPS technique reduces the number of phase shifters for linear [21] and planar [22] antenna arrays. Although these configurations of the previous work provide an interesting way of applying CORPS considering the cophasal excitation required for beam-scanning, the application of CORPS for reducing the number of phase shifters in the beam-forming network is really scarce in the literature. The CORPS concept can be considered to design new schemes or methodologies that simplify the complexity of the antenna system with the required radiation characteristics.

This paper introduces a new scheme of applying CORPS for reducing the number of phase shifters in linear antenna arrays. This new scheme applies an interleaving of several blocks of 2 × 3 CORPS networks. The outputs of a set of 2 × 3 CORPS networks are connected to the inputs of other set of 2 × 3 CORPS networks. This interleaving of two stages of 2 × 3 CORPS networks is made in a convenient way to provide the required progressive phase for beam-scanning and the level of amplitude excitations necessary for achieving the radiation characteristics of low SLL. In this case, a raised cosine amplitude distribution is applied to obtain low SLL. Interesting results are provided based on experimental measurements and full-wave simulations to analyze and evaluate the performance of the feeding network based on CORPS and the reduction capability of the number of phase shifters in the antenna system. The proposed design methodology achieves a reduction capability of 66% in the number of phase shifters used in linear antenna arrays. This reduction in the complexity of the antenna system is reached maintaining a low value of SLL with scanning ranges of until ±25°. The feeding network and the full system using linear antenna arrays were validated using experimental measurements and electromagnetic simulations. This proposed technique provides several benefits with respect to other techniques in the literature.

## 2. Proposed Array Design Methodology

An interesting scheme to feed linear antenna arrays is proposed to achieve a reduction in the number of phase shifters in the system. So, the model and the proposed design configuration is described for this geometry. This new scheme can be seen as a combination of properties of two techniques: CORPS and crossovers.

### 2.1. Theoretical Aspects of CORPS

**Figure 1:**
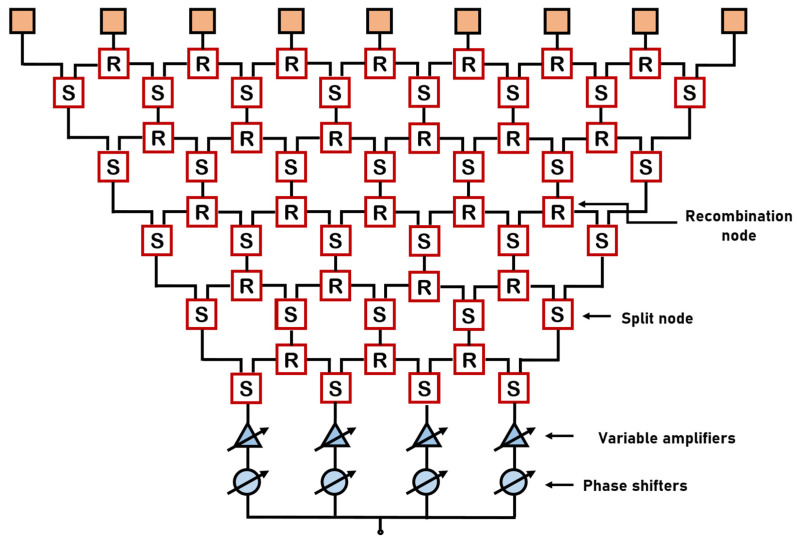
Standard scheme for a CORPS feeding network of 9 elements and 4 input ports.

The feeding network systems based on CORPS have been introduced previously in [20,21,22,23]. The CORPS networks consist of the iteration of recombination and split nodes [23]. The key theoretical aspect of these networks is the power or energy propagation through the network using these nodes (split or recombination) [23]. Figure 1 illustrates this; it shows a linear antenna array using 9 elements and a CORPS feeding network of 5 layers and 4 input ports. Then, the signals set at the input ports are split and recombined in each layer delivering a phase and amplitude distribution at the output ports. Amplifiers and phase shifters can be used at the input ports to generate desirable characteristics of the phase and amplitude at the output ports. The basic case of Figure 1 reduces from 9 phase shifters (used in a traditional phased array) to 4. As set in [23], the design configuration of these networks must consider that the losses increases if the number of layers increases.

**Figure 2:**
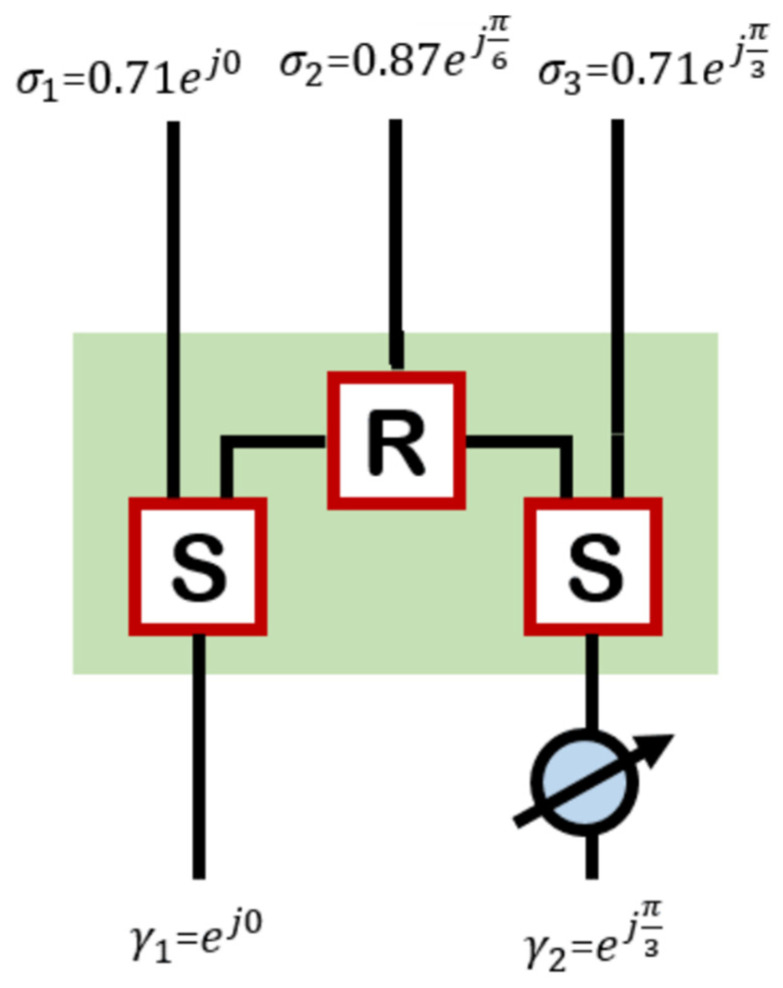
CORPS feeding network of 2 input ports and 3 output ports.

An interesting property of the CORPS networks was studied in [21,22]. This property considers that if 2 signals are fed to a CORPS network of 2 × 3, as shown in Figure 2, the phase average of the two signals is obtained at the output of the recombination node. This property is useful to generate the desirable progressive phase for linear antenna array configurations [21].

The proposed scheme takes advantage of using blocks of 2 × 3 CORPS networks and certain crossovers to interconnect two stages of 2 × 3 CORPS networks in a convenient way to provide the required progressive phase for beam-scanning. Though the crossovers in the design of antenna arrays could be undesirable, the crossovers can be useful to set new and different configurations by interconnecting different stages or blocks of 2 × 3 CORPS networks. The combination of these two properties helps to set a new configuration for reducing the number of phase shifters.

### 2.2. Design Configuration for Linear Antenna Arrays

**Figure 3:**
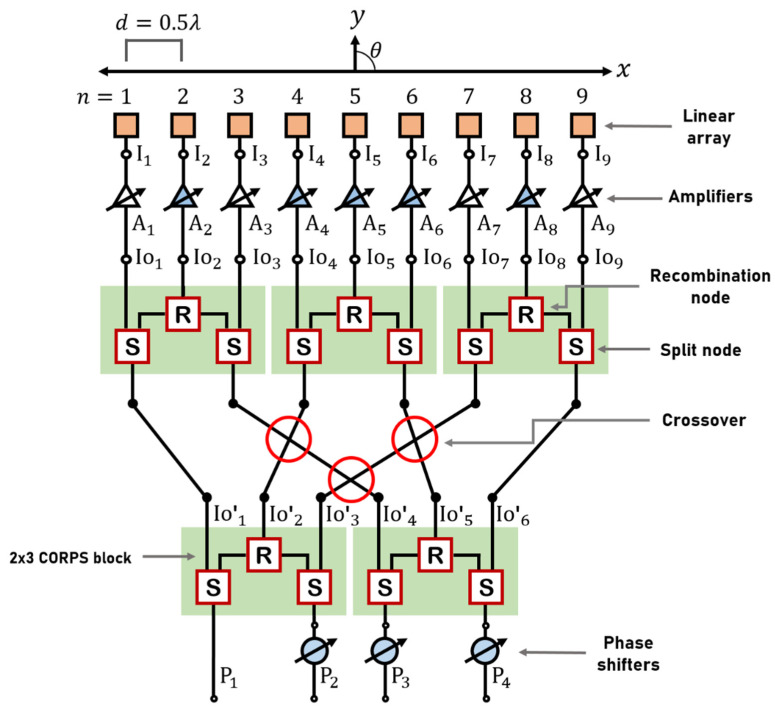
Proposed design scheme for a linear antenna array of 9 elements based on 2 × 3 CORPS networks and crossovers.

The description of this proposed design scheme begins setting the array factor for the linear antenna array of 9 elements, as shown in Figure 3. The array factor is given as a function of *θ* using the next equation [24]:

(1) $$AF\left(\theta \right)=\overset{N}{\underset{n=1}{\sum }}I_{n}e^{j\left(kd\left(n-1\right)sin\left(\theta \right)+\alpha_{n}\right)}$$

*N* is the number of antenna elements; each antenna element has an amplitude excitation using a fixed or variable amplifier and $I_{n}$ is the *n*th amplitude excitation, *d* is given as the separation between antenna elements, k is the phase constant, and $\alpha_{n}$ is the progressive phase excitation for beam-steering at *θ*_0_. The progressive phase excitation is calculated as:

(2) $$\alpha_{n}=-kd\left(n-1\right)\sin\left(\theta_{0}\right)\;n=1,\;2,\;\ldots ,\;N\;$$

Then, a progressive phase excitation is required at the antenna elements. Therefore, before the amplifiers stage, each group of three antenna elements is fed by a 2 × 3 CORPS network. As demonstrated in [21], one 2 × 3 CORPS network provides the average value of the phase values received at its two input ports. This network configuration applies an interleaving of two stages of 2 × 3 CORPS networks. The outputs of two 2 × 3 CORPS networks (first stage) are connected to the inputs of three 2 × 3 CORPS networks (second stage) to feed the 9 elements of the linear array (Figure 3). If we follow the phase values at each stage of the proposed network from input ports (*P*_1_, *P*_2_, *P*_3_ and *P*_4_), the required progressive phase for beam-scanning can be set at the antenna elements of the array system. The phase values *P*_1_, *P*_2_, *P*_3_, and *P*_4_ are set at the input ports in a convenient way to consider the required phase values. These required phase values can be generated from Equation (2) for a desired value of *θ*_0_. The input port 1 does not have a phase shifter. This is because the phase value *P*_1_ = 0 for all scanning directions.

A raised cosine distribution is generated for the amplitude excitations at the antenna elements to obtain low SLL values. Variable amplifiers are considered at the outputs from a recombination node and fixed amplifiers from split nodes. The center of the linear array is the phase reference, and *d_n_* can be set as the distance of the *n*th element to the antenna array center. The distribution of raised cosine for amplitude excitations can be calculated using the next equation [1]:

(3) $$I_{n}=\frac{1+\cos\left(\frac{d_{n}cos^{-1}\left(2a-1\right)}{0.5L}\right)}{2}\;n=1,\;2,\;\ldots ,\;N\;$$

In the last expression, the value of *a* can be considered fixed and *L* as the array longitude. A value of *a* = 0.35 can be set to reach a desired SLL of −22 dB in a scanning range of ±25°. The amplitude excitations values (*I_n_*) must be determined considering the level of amplitude values at the output ports (*IO_n_*) of the 4 × 9 CORPS network.

The required amplification values at the outputs of the feeding network (*A*_1_…*A*_9_) for different scanning directions are shown in Table 1. The required value at the output of a recombination node is higher for higher scanning values.

The proposed scheme can be designed considering the higher value of antenna elements in the linear array by adding more input ports (and 2 × 3 CORPS networks). Table 2 shows a behavior of the numerical values (array factor performance) that could be reached as the number of antenna elements increases. Table 2 indicates that performance could be maintained in the reduction of phase shifters and SLL (below −20 dB).

### 2.3. Proposed Feeding System Based on 4 *×* 9 CORPS Network

**Figure 4:**
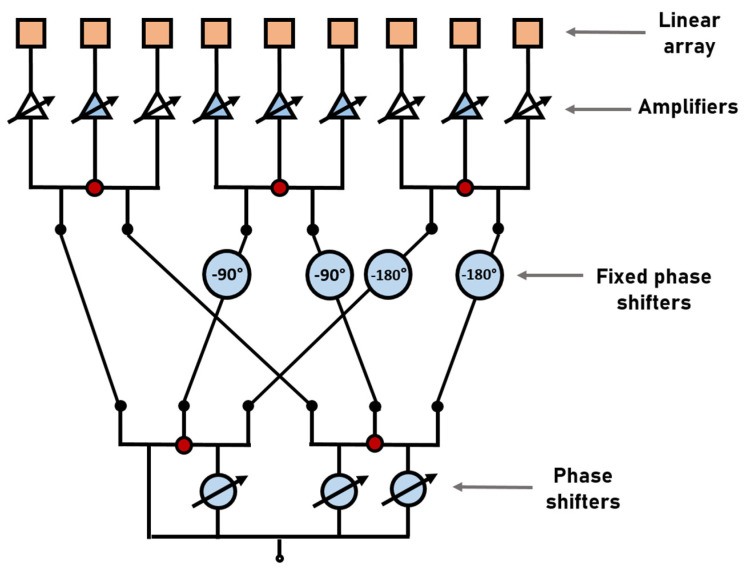
Scheme to illustrate the recombination nodes for the 4 × 9 CORPS network.

As seen previously, the proposed scheme for linear phased arrays is based on a 4 × 9 CORPS network to provide the progressive phase for beam-scanning and to reduce the number of phase shifters. In comparison with a conventional CORPS [20], the proposed feeding system can achieve a good control of phase for beam-scanning and the number of recombination nodes (losses by energy dissipation) is lower, as observed in Figure 4. A total of 25 recombination nodes are used in a conventional CORPS network (a CORPS network of 4 inputs and 9 outputs with 5 layers), and 5 recombination nodes for the proposed feeding system. This means a reduction of 80% of recombination nodes. Fonseca et al. [25] advises a value of phase difference within the range 0° to 90° (between the two input ports of each 2 × 3 CORPS network) to avoid recombination losses. Therefore, we tried to avoid recombination losses by taking this consideration into account using fixed phase shifters of 90° and 180°, as indicated in Figure 4. Then, the use of these fixed phase shifters helps to set phase differences within the range 0° to 90° at the input ports of 2 × 3 CORPS networks of the first stage. The values of the fixed phase shifters compensate this phase difference, avoiding severe recombination losses. Furthermore, this is helpful to generate the amplitude excitation values of the raised cosine distribution. These amplitude excitation values are less attenuated by using the fixed phase shifters illustrated in Figure 4.

A mathematical description of the 4 × 9 CORPS network can be given by considering the next scattering matrix of each 2 × 3 CORPS network [20]:

(4) $${\left[S\right]}^{NETWORK}=\left[\begin{array}{l} \frac{1}{\sqrt{2}}\;0\\ \frac{1}{2}\;\;\frac{1}{2}\\ 0\;\frac{1}{\sqrt{2}} \end{array}\right]$$

Each 2 × 3 CORPS network has two inputs and three outputs. The complex inputs can be named as *a*_1_ and *a*_2_. The outputs (*b*_1_ *b*_2_ *b*_3_) of each 2 × 3 CORPS network are generated by multiplying the complex inputs by the matrix of the Equation (4), and we have as a result $\frac{a_{1}}{\sqrt{2}}$, *a*_1_/2 + *a*_2_/2 and $\frac{a_{2}}{\sqrt{2}}$**.**

Therefore, we can follow each stage of the proposed feeding network to see the values of amplitude and phase generated by the complex inputs; Figure 3 illustrates this. The complex inputs are named *P*_1_, *P*_2_, *P*_3_, and *P*_4_. If we analyze the first stage, the first 2 × 3 CORPS network has the inputs *P*_1_ and *P*_2_, and the second 2 × 3 network has the inputs *P*_3_ and *P*_4_. Therefore, it is easy to determine that the outputs of the first stage are:

(5) $${\mathrm{IO}}_{1^{\prime }}=\frac{P_{1}}{\sqrt{2}}$$

(6) *IO*_2′_ = *P*_1_/2 + *P*_2_/2

(7) $${\mathrm{IO}}_{3^{\prime }}=\frac{P_{2}}{\sqrt{2}}$$

(8) $${\mathrm{IO}}_{4^{\prime }}=\frac{P_{3}}{\sqrt{2}}$$

(9) *IO*_5′_ = *P*_3_/2 + *P*_4_/2

(10) $${\mathrm{IO}}_{6^{\prime }}=\frac{P_{4}}{\sqrt{2}}$$

By following the structure of the proposed feeding network (Figure 3), the outputs of the first stage (*IO*_1′_, *IO*_2′_, *IO*_3′_, *IO*_4′_, *IO*_5′_, and *IO*_6′_) are the inputs of the second stage, i.e., the left 2 × 3 network has the inputs *IO*_1′_ and *IO*_4′_, the central 2 × 3 network has the inputs *IO*_2′_ and *IO*_5′_, and the right 2 × 3 CORPS network has *IO*_3′_ and *IO*_6′_ as inputs. Then, the outputs of the second layer are:

(11) $${\mathrm{IO}}_{1}=\frac{IO_{1^{\prime }}}{\sqrt{2}}$$

(12) *IO*_2_ = *IO*_1′_/2 + *IO*_4′_/2

(13) $${\mathrm{IO}}_{3}=\frac{IO_{4^{\prime }}}{\sqrt{2}}$$

(14) $${\mathrm{IO}}_{4}=\frac{IO_{2^{\prime }}}{\sqrt{2}}$$

(15) *IO*_5_ = *IO*_2′_/2 + *IO*_5′_/2

(16) $${\mathrm{IO}}_{6}=\frac{IO_{5^{\prime }}}{\sqrt{2}}$$

(17) $${\mathrm{IO}}_{7}=\frac{IO_{3^{\prime }}}{\sqrt{2}}$$

(18) *IO*_8_ = *IO*_3′_/2 + *IO*_6′_/2

(19) $${\mathrm{IO}}_{9}=\frac{IO_{6^{\prime }}}{\sqrt{2}}\;$$

**Figure 5:**
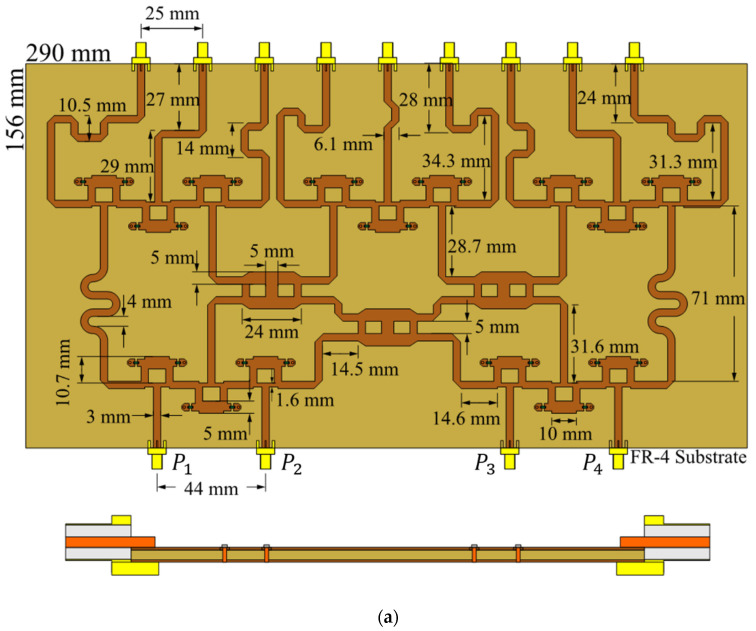

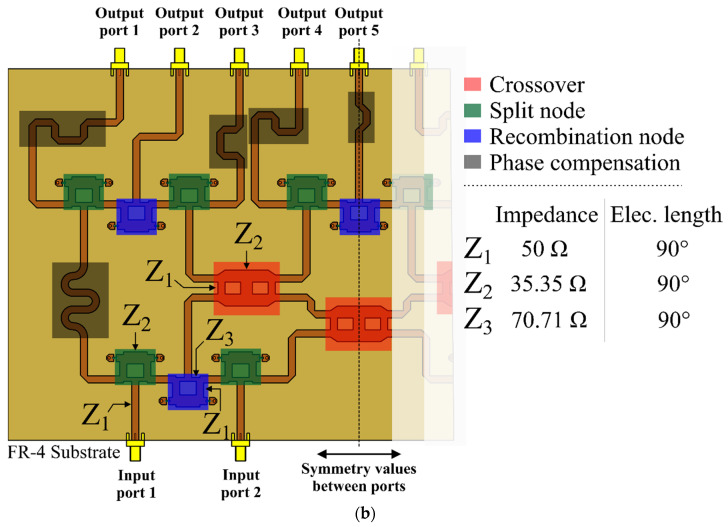
Design of the proposed feeding system based on 4 × 9 CORPS network: (**a**) Design in CST Microwave Studio, (**b**) components and values of the characteristic impedances of all transmission line sections.

Then, the proposed feeding scheme can be designed for studying its behavior and performance. Figure 5 shows the design in CST Microwave Studio of the proposed feeding system. The feeding system in Figure 5 considers a frequency at 6 GHz using Gysel power dividers [26] and resistances of surface mount (50 Ohms-FC0603). The Gysel power dividers operate as split or recombination nodes. Furthermore, a substrate of 290 × 156 mm is considered with the next characteristics: FR4, thick = 1.6 mm, relative permittivity 𝜀𝑟 = 4.2, tangent loss = 0.025, and 𝜇 = 1.0. The values of length and width of the transmission line sections are illustrated in Figure 5a). Figure 5b) indicates where the components are located and the values of the characteristic impedances of all transmission line sections. The process of simulation (full-wave CST) takes the resistances and the SMA connectors into account.

The system of 9 antenna elements of the linear array are controlled with 3 input ports (or phase shifters), as illustrated in Figure 5. The nine phase values (generated by a conventional system of progressive phase excitation) are generated using only three control ports (3 phase shifters). The phase values at the input ports (*P*_1_, *P*_2_, *P*_3_ and *P*_4_) are set in according to the phase values required at the antenna elements: 1, 3, 7, and 9 (Equation (2)). Then, the blocks of 2 × 3 CORPS networks and the crossovers provide the phase values required at the antenna elements 2, 4, 5, 6, and 8. That is the key stone of the proposed feeding system. The crossovers are designed in a conventional way by considering line couplers as detailed in [27,28,29].

## 3. Results and Discussion

The antenna system of the linear phased array was evaluated considering the experimental measurements of the proposed feeding system based on 4 × 9 CORPS network. The details of the prototype and experimental measurements are given in the next sections.

### 3.1. Measurements of the Proposed Feeding System (4 *×* 9 CORPS Network)

**Figure 6:**
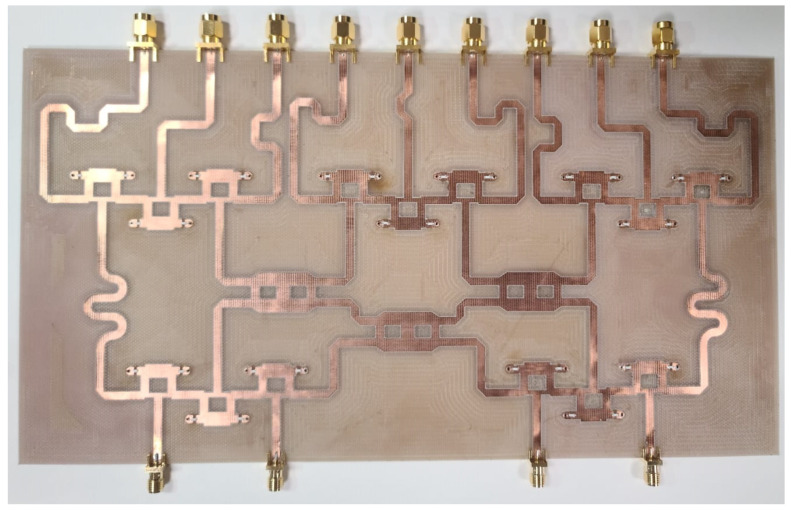
Fabricated prototype of the proposed feeding system.

**Figure 7:**
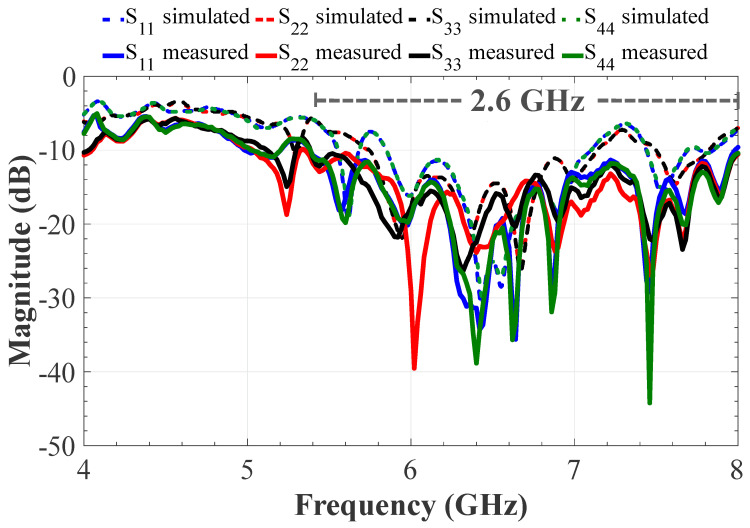
Reflection coefficients simulated and measured for the proposed feeding system.

The proposed feeding system of the 4 × 9 CORPS feeding network was fabricated and measured to evaluate its performance and its impact in feeding linear phased arrays. Figure 6 illustrates the fabricated prototype using the design values given in the previous section. Figure 7 illustrates the reflection coefficients simulated by CST and measured experimentally for the proposed feeding system of the 4 × 9 CORPS network. The measurement results gives a bandwidth of 2.6 GHz for the proposed feeding system, as shown in Figure 7. The measurement results show that the maximum value of the reflection coefficients is approximately −17 dB at 6 GHz (set as the design frequency). Figure 7 indicates that the behavior of the results of the reflection coefficients obtained by simulation and experimental measurements are very similar.

**Figure 8:**
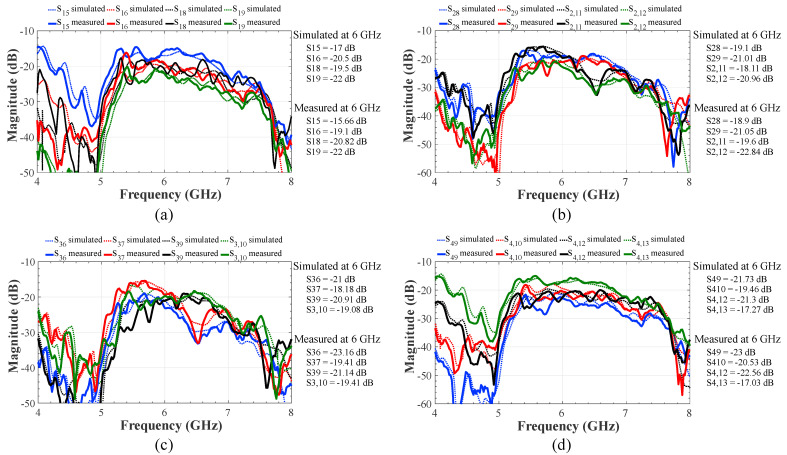
Transmission coefficients simulated and measured experimentally for the proposed feeding system (of 4 × 9 CORPS network) for each input port: (**a**) P1, (**b**) P2, (**c**) P3 and (**d**) P4.

Figure 8 illustrates the transmission coefficients simulated by CST and measured experimentally for the proposed feeding system. Power losses are generated by Gysel power dividers, SMA connectors, crossovers, and energy dissipation. However, the measurement results shows a good transmission considering the output ports of interest for each input port. The numbers 5, 6, 7, 8, 9, 10, 11, 12, and 13 indicate the output ports 1, 2, 3, 4, 5, 6, 7, 8, and 9 of the feeding system (4 × 9 CORPS network). As commented previously, a variable amplifier is considered at each output from a recombination node of the proposed feeding network [21]. The electromagnetic simulation results and the measurement results are very similar.

### 3.2. Experimental Evaluation of the Linear Phased Array

The measured CORPS feeding network (4 × 9) was used to feed the linear phased array considering the performance in the reduction of phase shifters and scanning capabilities. A separation of *d* = 0.5λ is considered as uniform in the array system. The linear phased array was designed, simulated electromagnetically, and fabricated to analyze its performance considering the error of amplitude and phase of the proposed feeding system (4 × 9 CORPS network). A circular patch is considered at a central frequency of 6 GHz with the next design characteristics: *r* = 13.02 mm, *h* = 1.6 mm (FR4 substrate) and =2.07 [24]. The radiation efficiency is approximately 90% considering the FR4 substrate for the design frequency.

**Figure 9:**
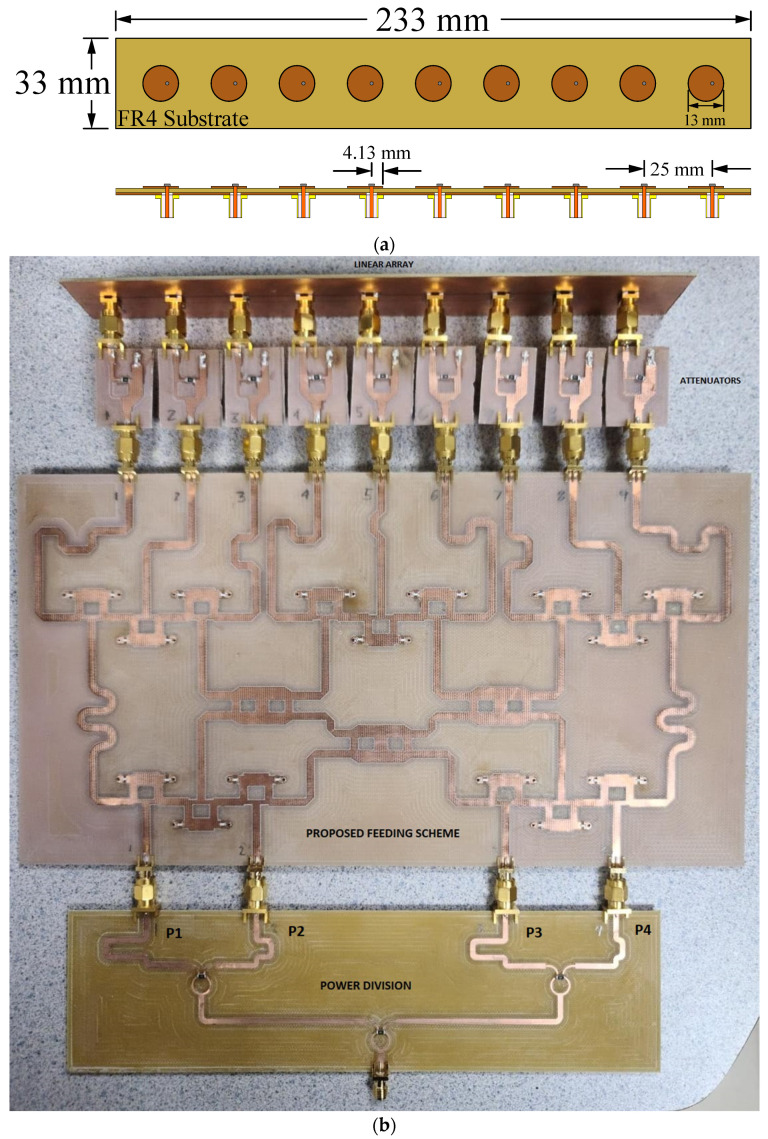
Linear phased array, (**a**) antenna elements configuration, and (**b**) the fabricated prototype of the complete system with power values of approximately 15 dBm, and phase shift values of (0°, 152.14°, 96.42°, 248.57°) for *P*_1_, *P*_2_, *P*_3_ and *P*_4_.

**Figure 10:**
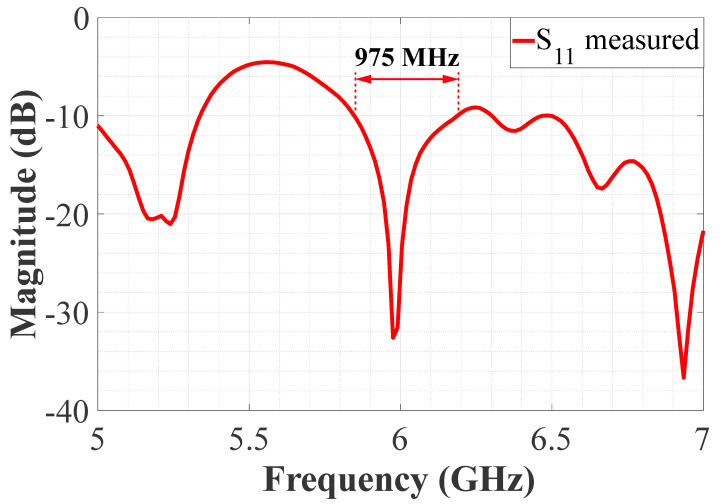
Reflection coefficient for the complete system measured by a vector network analyzer.

**Figure 11:**
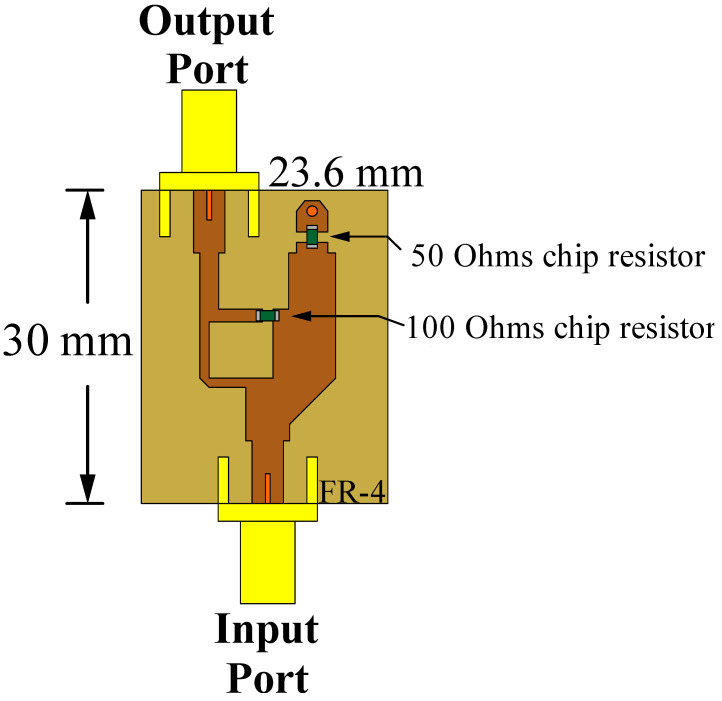
Example of the schematic diagram for the attenuators.

The fabricated prototype of the complete system for the linear phased array is illustrated in Figure 9. The complete array system includes the antenna elements (set as shown in Figure 9a), the stage of attenuators, the proposed feeding network (4 × 9), and the power division network. Every stage was fabricated on FR4. The reflection coefficient for the entire system was measured in a vector network analyzer, and its behavior is shown in Figure 10. As illustrated in this figure, each stage was designed and fabricated to match the design requisites. The system accomplishes the impedance requirements to operate in a bandwidth of 975 MHz (considering 6 GHz as the design frequency). The stage of attenuators was designed and fabricated using the technique of unequal Wilkinson power division [30]. In this case, resistors of surface mount (50 and 100 Ohms) are considered as shown in Figure 11. Transmission lines (of different length) are employed as the phase shifts. The proposed feeding network is fed by the power divider. Then, the outputs of the proposed feeding network are connected to the stage of attenuators and antenna elements.

The proposed feeding system using the linear phased array generates a reduction of phase shifters, providing a maximum SLL of approximately −22 dB for the range of 𝜃_0_ = ±25°. The SLL performance (−22 dB) is maintained during beam-scanning in 𝜃_0_ = ± 25°. 

**Figure 12:**
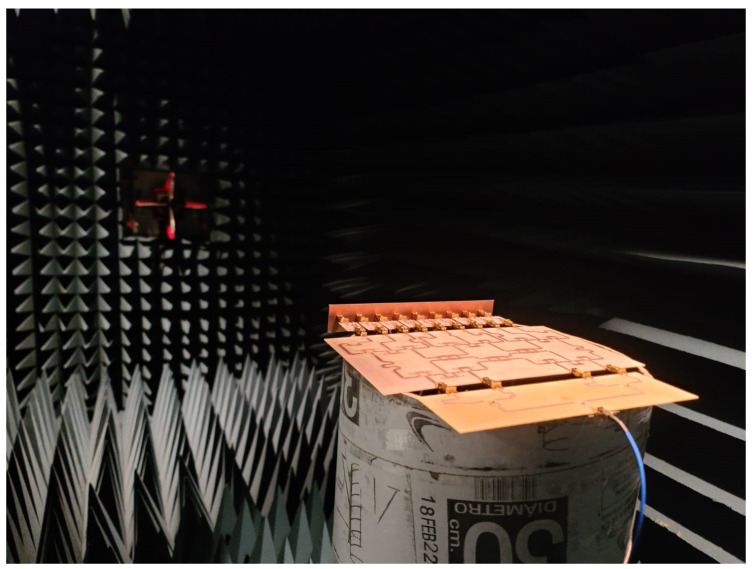
Prototype of the full system measured experimentally in a far field anechoic chamber.

**Figure 13:**
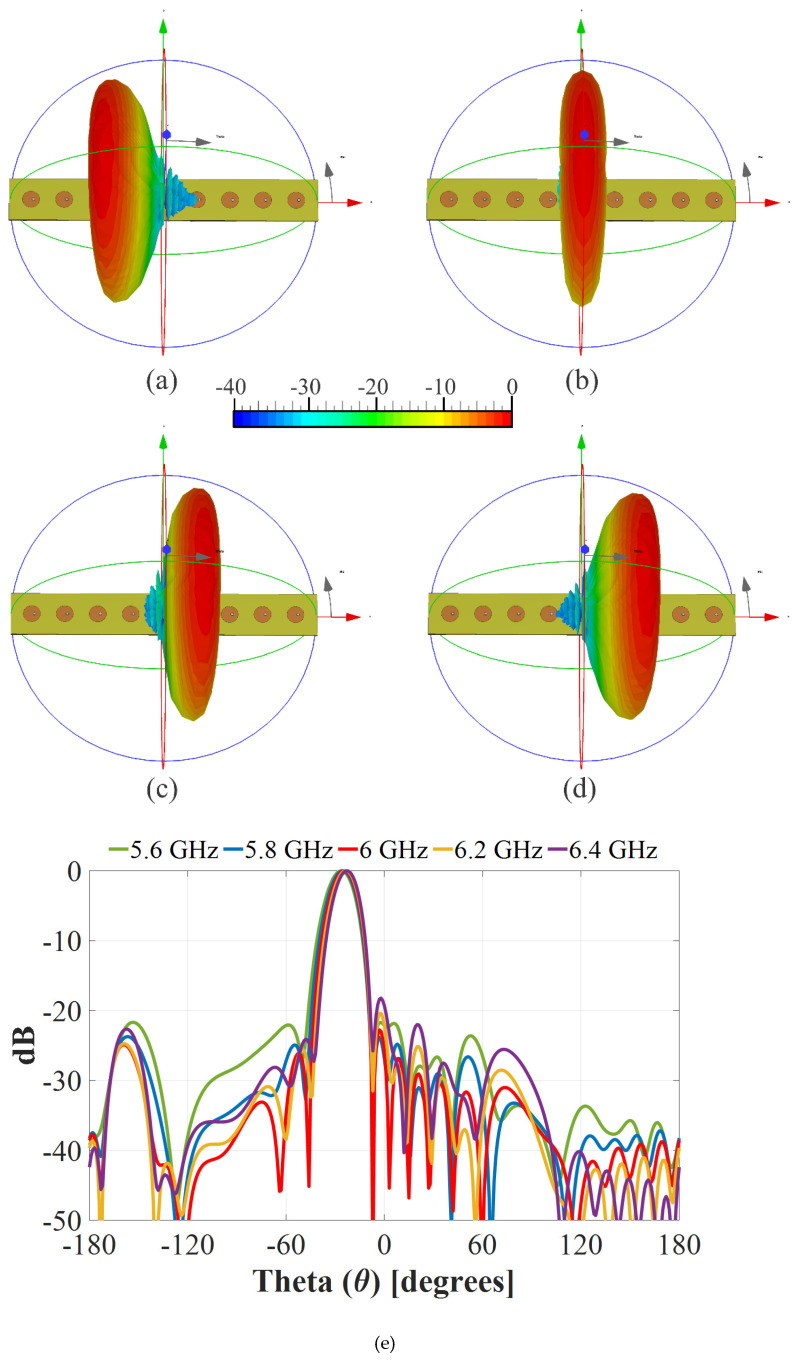
Radiation pattern obtained by CST simulations for (**a**) 𝜃_0_ = −25°, (**b**) 𝜃_0_ = 0°, (**c**) 𝜃_0_ = 15°, and (**d**) 𝜃_0_ = 25° and (**e**) measured experimentally for the complete system.

The radiation pattern for the prototype of the full system was measured experimentally in an anechoic chamber (far field), as shown in Figure 12. Furthermore, the complete system based on the linear array was simulated using full wave software (CST) to study the behavior of the radiation pattern generated. Figure 13 shows the radiation pattern generated by electromagnetic simulations in CST for (a) 𝜃_0_ = −25°, (b) 𝜃_0_ = 0°, (c) 𝜃_0_ = 15°, and (d) 𝜃_0_ = 25° and (e) measured experimentally for the complete system of the linear phased array. Figure 13e illustrates the behavior of the radiation pattern (normalized) for the frequency values of 5.6 GHz, 5.8 GHz, 6 GHz, 6.2 GHz, and 6.4 GHz. This figure shows the radiation pattern for the farthest scanning direction (*θ*_0_ = −25°). There are some very slight changes in the radiation pattern for these frequency values. However, the radiation performance remains for these frequency changes. The use of the raised cosine distribution in the proposed design methodology generates low values of SLL (aprox. −19 dB) in the scanning range, as shown in the obtained results.

Table 3 illustrates a comparative analysis of the proposed feeding system with respect to other design cases based on linear array geometries. The proposed design methodology reduces the number of phase shifters by 66%. The proposed technique outperforms to other techniques in the reduction of the number of phase shifters to be used in the antenna system. This reduction capability in the number of phase shifters is reached while maintaining a low SLL in the scanning range of ±25°.

The values presented in Table 3 are based on the designs presented in each reference cited. Each paper or work provides the information of reduction of phase shifters, and we compared this information with that of our design case. This Table indicates the number of antenna elements used in the systems of each previous work. However, the reduction of phase shifters of each case in the table could be applicable if the number of elements is increased.

The technique presented in this paper can be considered a good design option to reduce the complexity of the feeding network in antenna array applications.

## 4. Conclusions

This paper presented a design scheme to feed linear antenna arrays and achieve reduction capability in the number of phase shifters of the system. A feeding network (4 inputs and 9 outputs) based on CORPS technology and crossovers was proposed. This proposed feeding network was designed, simulated, and implemented. The proposed feeding system uses only 5 recombination nodes, i.e., a reduction of 80% of recombination nodes with respect to a conventional CORPS network. Experimental measurements showed that the proposed feeding network can operate in a wide bandwidth and provide the progressive phase for beam-scanning while obtaining a reduction in the number of phase shifters employed.

The proposed feeding system was analyzed and evaluated to feed linear phased arrays with respect to other techniques. The obtained benefits were a reduction capability of 66% in the number of phase shifters and a good SLL performance for beam-scanning using a raised cosine distribution. The electromagnetic simulation results for the full system generate a radiation pattern with a scanning range of ±25° maintaining a maximum SLL of −22 dB during beam-scanning. Furthermore, the linear phased array was validated by taking measurements obtaining a slight deviation in the SLL (approximately −19 dB) for different frequency values.

## Figures and Tables

**Table 1:** Amplification values (at the outputs of the feeding system) that are required to obtain a raised cosine.

$\theta_{0}$	$A_{1}$	$A_{2}$	$A_{3}$	$A_{4}$	$A_{5}$	$A_{6}$	$A_{7}$	$A_{8}$	$A_{9}$
−5°	0.71	0.85	1.59	1.83	1.42	1.34	1.59	0.85	0.71
0°	0.71	0.82	1.59	1.33	1.00	1.33	1.59	0.82	0.71
5°	0.71	0.85	1.59	1.83	1.42	1.34	1.59	0.85	0.71
10°	0.71	0.96	1.59	1.34	1.17	1.34	1.59	0.96	0.71
15°	0.71	1.20	1.59	1.75	1.91	1.75	1.59	1.20	0.71
20°	0.71	1.73	1.59	1.34	2.11	1.34	1.59	1.73	0.71
25°	0.71	3.42	1.59	2.01	6.23	2.01	1.59	3.42	0.71

**Table 2:** Numerical values as the number of antenna elements increases.

Number of Elements	Number of Phase Shifters	Number of Fixed Phase Shifters	Number of 2 × 3 CORPS	SLL [dB]	Maximum Beamwidth
9	3	4	5	−22.64	34.41°
18	7	8	10	−20.73	16.39°
27	11	12	15	−20.89	10.63°
36	15	16	20	−20.39	8.11°
45	19	20	25	−20.55	6.31°
54	23	24	30	−20.38	5.23°
63	27	28	35	−20.49	4.51°

**Table 3:** A comparative analysis of the proposed feeding system with respect to other design cases based on linear array geometries.

	Number of Elements	Number of Phase Shifters	Reduction of Phase Shifters	Scanning Range	Peak Side Lobe Level	Number of Variable Amplifiers
This work	9	3	66%	±25°	−22 dB (sim.) −19 dB (mea.)	5
[21]	7	3	57%	±25°	−20 dB	3
[20]	10	8	20%	±30°	−19 dB	8
[1]	30	12	60%	±12°	−15 dB	12
[2]	28	14	50%	±24°	−15 dB	14

## Data Availability

Not applicable.
